# The first complete chloroplast genome of a major mangrove species *Sonneratia alba* Sm. and its implications on conservation efforts

**DOI:** 10.1080/23802359.2018.1463828

**Published:** 2018-04-23

**Authors:** Tianhui Yu, Damien Daniel Hinsinger, Joeri Sergej Strijk, Alison Kim Shan Wee

**Affiliations:** a State Key Laboratory for Conservation and Utilization of Subtropical Agro-bioresources, Guangxi University, Nanning, Guangxi, China;; b Biodiversity Genomics Team, Plant Ecophysiology and Evolution Group, Guangxi Key Laboratory of Forest Ecology and Conservation, College of Forestry, Guangxi University, Nanning, Guangxi, China;; c Ecological Genomics Team, Plant Ecophysiology and Evolution Group, Guangxi Key Laboratory of Forest Ecology and Conservation, College of Forestry, Guangxi University, Nanning, Guangxi, China

**Keywords:** Coastal, complete chloroplast genome, forest, Lythraceae, Myrtales

## Abstract

*Sonneratia alba* Sm. is one of the most widely distributed mangrove species worldwide. In this study, the whole chloroplast genome of *S. alba* was assembled for the first time not only in *Sonneratia*, but also for a member of the mangrove plant community. The total chloroplast genome was 153,061 bp in length, with a large single copy (LSC) region of 87,226 bp and a small single copy (SSC) region of 18,033 bp, separated by two inverted repeats (IRs) regions of 23,901 bp. The overall GC content was 37.3%, and 43.1%, 35.4%, and 31.1% in the IRs, LSC, and SSC regions, respectively. It contained 106 genes, including 79 coding genes, 24 tRNA genes, and four rRNA genes. A phylogenetic analysis confirmed that *S. alba* was clustered with *Trapa maximowiczii* within the family Lythraceae.

Mangroves are a taxonomically diverse group of plants that have undergone genome-wide convergent evolution to adapt to coastal conditions with fluctuating salinity and inundation (Xu et al. 2017). The genus *Sonneratia*, one of the major components of mangrove ecosystems, comprises six species distributed from East Africa to the West Pacific Ocean (Spalding et al. 2010), with *S. alba* being the most widely distributed species. Due to the overlapping distribution of congeneric species, natural hybridization is common in *Sonneratia* (Zhou et al. 2005). At the same time, the genus contains rare species of high conservation status; *S. griffithii* and *S. ovata* are listed as critically endangered and near threatened on the IUCN Red List, respectively (Duke et al. 2010; Salmo et al. 2010). Natural hybridization of *S. alba* and *S. griffithii* (Qiu et al. 2008) could threaten the species integrity of the latter and complicate current conservation efforts. Phylogeographic studies on *S. alba* revealed low genetic diversity in this species, especially at the outer edge of its distribution range, suggesting genetic erosion from repeated range contraction and expansion (Wee et al. 2017; Yang et al. 2017). Here, we report the complete chloroplast genome of *S. alba* to provide genomic resources for conservation and for investigating convergent evolution of mangrove species (e.g. Xu et al. 2017).

Leaf material was collected from one individual of *S. alba* (sahn1) in Hainan province (19° 37′ 40″, 110° 50′ 22″; China; voucher AKSW_SACH01 deposited in the herbarium of the Biodiversity Genomics Team (BGT), Guangxi University, Nanning, China). Genomic DNA extraction followed by library construction and sequencing on the Illumina hiSeq2500 (genome skimming) was performed by Novogene (Beijing, China). The chloroplast genome was assembled *de novo* using org.asm (ORG.ASM. 2016) and annotated using cpGAVAS (Liu et al. 2012).

The chloroplast genome of *S. alba* (GenBank accession MH105772) was 153,061 bp in length, consisting of a large single copy (LSC) with 87,226 bp, a small single copy (SSC) region of 18,033 bp, and a pair of 23,901 bp and 23,900 bp inverted repeats (IRs). The GC content in the chloroplast genome was 37.7% in the IRs, which was greater than that of the LSC (35.4%) and SSC (31.1%) regions. The genome contained 106 genes, including 79 coding genes, 24 tRNA genes, and four rRNA genes.

This is the first chloroplast genome to be published for *Sonneratia* and for major mangrove species. We compared the phylogenetic relationship of *S. alba* with other species from the Myrtales using sequences obtained from GenBank. The phylogenetic tree was constructed using phyML 3.0 with 100 bootstrap repeats (Guindon et al. 2005). The resulting tree supported the monophyly of the Lythraceae and the sister relationship between *Sonneratia* and *Trapa* (Figure 1). The evolutionary relationships of Lythraceae, Onagraceae, Myrtaceae, and Melastomataceae are consistent with previously reported results (Kriebel et al. 2017).

**Figure 1.**
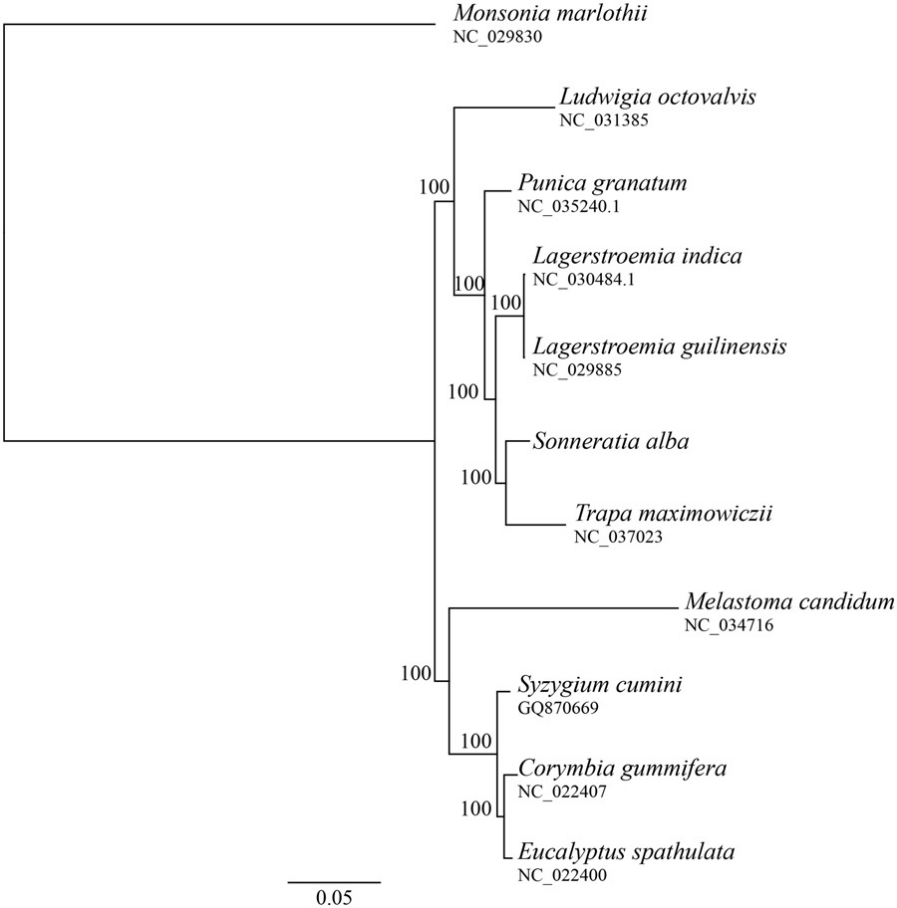
Maximum likelihood (ML) phylogenetic tree based on complete chloroplast genome sequences of *Sonneratia alba* and other nine species from the order Myrtales using *Monsonia marlothii* of Geraniaceae as an outgroup. Numbers on branches are bootstrap support value based on 100 iterations.

The chloroplast genome of *S. alba* can be used to identify variable regions to study the phylogeography of its endangered congeneric species *S. griffithii* and the extent of natural hybridization between them. Furthermore, the chloroplast genome could also be used to investigate the extent of convergent evolution in photosynthetic genes among species frequently exposed to ecophysiological stress.
